# Anti-Osteoporotic Activity of an Edible Traditional Chinese Medicine *Cistanche deserticola* on Bone Metabolism of Ovariectomized Rats Through RANKL/RANK/TRAF6-Mediated Signaling Pathways

**DOI:** 10.3389/fphar.2019.01412

**Published:** 2019-11-26

**Authors:** Bo Zhang, Ling-Ling Yang, Shu-Qin Ding, Jing-Jing Liu, Yan-Hong Dong, Yan-Ting Li, Nan Li, Xiao-Jun Zhao, Chang-Ling Hu, Yiping Jiang, Xue-Qin Ma

**Affiliations:** ^1^Key Laboratory of Hui Ethnic Medicine Modernization, Department of Pharmaceutical Analysis, Ministry of Education, School of Pharmacy, Ningxia Medical University, Yinchuan, China; ^2^Laboratory for Functional Foods and Human Health, Center for Excellence in Post-Harvest Technologies, North Carolina Research Campus, North Caroline A&T State University, Greensboro, NC, United States; ^3^Department of Pharmacognosy, The Second Military Medical University, Shanghai, China

**Keywords:** *Cistanche deserticola*, antiosteoporotic, TRAF6, RANKL, RANK

## Abstract

Given the limitations of existing therapeutic agents for treatment of postmenopausal osteoporosis, there still remains a need for more options with both efficacy and less adverse effects. *Cistanche deserticola* Y. C. Ma is known as a popular tonic herb traditionally used to treatment deficiency of kidney energy including muscle weakness in minority area of Asian counties. Based on the theory of “kidney dominate bone,” an ovariectomized (OVX) rat model of postmenopausal osteoporosis was used to evaluate the therapeutic effect of *C. deserticola* extract (CDE) on bone loss. Forty eight female Sprague-Dawley rats, aged about 12 weeks, were randomly assigned into six groups including sham group orally administrated with 0.5% carboxymethyl cellulose sodium (CMC-Na) (sham), positive group treated with 1 mg/kg of estradiol valerate (EV), low, moderate, and high dosage groups orally administrated with 200, 400, and 800 mg/kg/day of CDE, respectively. After 3 months of continuous intervention, CDE exhibited significant anti-osteoporotic activity evidenced by the enhanced total bone mineral density, ameliorated bone microarchitecture; increased alkaline phosphatase activity; decreased deoxypyridinoline, cathepsin K, tartrate-resistant acid phosphatase, and malondialdehyde levels; whereas the body, uterus, and vagina weights in OVX rats were not influenced by CDE intervention. In addition, a seemed contradictory phenomenon on levels of calcium and phosphorus between OVX and sham rats were observed and elucidated. Mechanistically, CDE significantly down-regulated the levels of TRAF6, RANKL, RANK, NF-κB, IKKβ, NFAT2, and up-regulated the phosphatidylinositol 3-kinase (PI3K), AKT, osteoprotegerin, and c-Fos expressions, which implied CDE could suppress RANKL/RANK-induced activation of downstream NF-κB and PI3K/AKT pathways, and ultimately, preventing activity of the key osteoclastogenic proteins NFAT2 and c-Fos. All of the data suggested CDE possessed potential anti-osteoporotic activity and this effect was, at least in part, involved in modulation of RANKL/RANK/TRAF6-mediated NF-κB and PI3K/AKT signaling as well as c-Fos and NFAT2 levels. Therefore, CDE may represent a useful promising remedy candidate for treatment of postmenopausal osteoporosis.

## Introduction

Since osteoporosis has not been regarded as part of normal aging, extensive advances in the osteoporosis field have been obtained. Today, osteoporosis has become a major health hazard afflicting more than 200 million populations all over the world with attendant 10 billion dollars were spent every year in these individuals (Ma et al., 2015). Osteoporosis, including postmenopausal and senile osteoporosis, was characterized by micro-architectural deterioration and low bone mass density (BMD) as a result of an imbalance between osteoblast mediated bone formation and osteoclast mediated bone resorption, respectively (Kanis et al., 2015). In consideration of the limitations of current therapeutic options and some unwanted adverse effects of synthetic agents (Ye et al., 2015) for treatment of osteoporosis disease, it is necessary for alternatives with both efficacy and minimal side effects (Tan et al., 2017). As a remarkable complementary and alternative therapy way for treatment of various aliments including postmenopausal osteoporosis, the natural medicinal herb especially traditional Chinese medicines (TCM) were gradually recognized and highly warranted (Czerwinska and Melzig, 2018; Ma et al., 2018).

Two thousands years ago, *Cistanche deserticola* Y. C. Ma was found beneficial for human health and recorded in the Chinese classical medicinal book named Shen Nong Herbal (Anonymous, 1981). Now, it is an important TCM officially recorded in the Chinese pharmacopoeia (Chinese and Pharmacopoeia, 2015) and also as a famous tonic herb which has been used in Asian counties including China and Japan for centuries of years (Wong et al., 2006). *C. deserticola* was found possessed a favorable safety profile (Liao et al., 2018) and broad medicinal functions: in folk, it was widely used to cook with kinds of sorts of herbal cuisine for the treatment of kidney deficiency, muscle debility, and lumbar weakness; nowadays, increasing attentions have been paid for its various pharmacological functions including anti-inflammatory, anti-fatigue, antitumor, antioxidant activity, enhancing immunity, and so on (Gu et al., 2016; Fu et al., 2018). It is also well known as “ginseng of the desert” in China due to its excellent clinical medical curative effect. Both the traditional and modern application of *C. deserticola* made this herb popular in both medicine and health food industry, thus had been developed into medicinal and nutritional liquid approved by the State Food and Drug Administration. Given *C. deserticola* was usually employed to deal with kidney deficiency in Chinese folk medicine, which implied this edible medicinal herb can be regarded as promising alternative agent to intervene osteoporosis based on the theory of “kidney dominate bone” (Wang et al., 2016). Published data had proved the anti-osteoporotic effect of *C. deserticola* extracts both *in vivo* and *in vitro* (Liang et al., 2011; Li et al., 2012; Liang et al., 2013; Xu et al., 2017; Song et al., 2018), and several isolated compounds including echinacoside (Li et al., 2013), acteoside (Lee et al., 2013), and cistanoside A (Xu et al., 2017) which also had been reported processing anti-osteoporotic activities; and other compounds like 2’-acetylacteoside was confirmed possessed antioxidant, anti-inflammatory, neuroprotective, hepatoprotective, immune-enhancing (Li et al., 2016), and anti-aging potentials (Peng et al., 2016). To date, dozens of bioactive phenylethanoid glycosides have been identified from *Cistanche* herb (Wang et al., 2012), echinacoside, and acteoside are the main compounds existing in most of the *Cistanche* species with the contents were 1.83–41.49 and 0.27–8.28 mg/kg, respectively; whereas the other phenylethanoid glycosides including 2’-acetylacteoside, 6’-acetylacteoside, cistanoside A, cistanoside C, and isoacteoside were 1.56–3.16 mg/g, 0.49–1.66 mg/kg, 1.41–10.11 mg/kg, 0.33–2.24 mg/kg, and 0.08–5.00 mg/kg, respectively (Dong et al., 2018). Based on the above publish data, we found that the underlying anti-osteoporotic molecular mechanisms of *C. deserticola* especially the upstream signaling was remain unclear; and the compound itself can not represent the effect of the herb which contained the synergy property contributed by different type of components; furthermore, the specific targets of signaling pathways were totally different between different components. The current work aimed at investigating the potential protective effect of *C. deserticola* against ovariectomized (OVX) rats *in vivo* with emphasis on the regulation of tumor-necrosis factor receptor-associated factor 6 (TRAF6), receptor activator of nuclear factor kappa B ligand (RANKL), RANK expressions and RANKL/RANK/TRAF6-induced nuclear factor kappa B (NF-κB), and phosphoinositide 3-kinase (PI3K)/protein kinase (AKT) signalings.

Bone remodeling, include reconstruction and repairment, is an intricate process that governed by the balance between osteoblastic bone formation and osteoclastic bone resorption, and an increased bone resorption mainly caused by enhanced osteoclastogenesis usually led to osteoporosis even bone fracture. Most of cell signals, hormones and growth factors which were essential for osteoclast differentiation and function were controlled by RANK and its two ligands, RANKL and osteoprotegerin (OPG) (Lacey et al., 1998). And recent findings further discovered TRAF6 was a key regulatory factor in RANKL/RANK triggered signaling cascades (Tan et al., 2017). By recruitment of TRAF6, RANKL bound to its receptor RANK, and then the downstream signaling cascades including NF-κB and PI3K/AKT were triggered, then the key osteoclastogenic proteins of c-Fos and nuclear factor of activated T cells c2 (NFAT2) (Takayanagi, 2007) were up-regulated, and finally the differentiation of osteoclast was initiated. In the present study, the underlying anti-osteoporotic mechanism of CDE on RANKL/RANK/TRAF6-mediated bone resorption is investigated and discussed.

## Materials and Methods

### Animals, Cells, Antibodies, and Reagents

Female adult Sprague-Dawley rats (Ningxia Medical University, Yinchuan, China); RAW264.7 cells (Zhong Qiao Xin Zhou Biotechnology Co., Ltd., Shanghai, China); macrophage colony-stimulating factor (M-CSF) and RANKL (PerroTech, Inc. USA); estradiol valerate [estradiol valerate (EV), Delpharm Lille SAS, Paris, France]; cathepsin K (CK) ELISA kit (BioVision, American); deoxypyridinoline (DPD) and bone gla-protein (BGP) cross-links ELISA kits (Xinyu Biological Engineering Co., Ltd, Shanghai, China); malondialdehyde (MDA), glutathione (GSH), and superoxide dismutase (SOD) reagent kits (Jianchen Biological Engineering, Nanjing, China); penicillin and streptomycin (Gibco, USA); polyvinylidene fluoride (PVDF) membrane (Millipore Life Sciences, USA); primary antibodies including GAPDH (18AF0401), β-actin (17AV0411), IκB kinase β (IKKβ) (AD01134589), TRAF6 (2), RANKL (GR3193842-5), RANK (AA02113656), NFκBIA (AH04138226), PI3K (AC09021266), OPG (AG06292776), c-Fos (AG12059411), AKT (AF05173234), NFAT2 (AO11015648), and secondary antibodies (horseradish peroxidase-conjugated goat anti-rabbit immunoglobulin G, 134658) were offered by ZSGB-BIO (Beijing, China); Dulbecco’s modified eagle’s medium (DMEM), bicinchoninic acid protein quantization assay kit, fetal bovine serum (FBS), and Dulbecco’s phosphate buffered saline (PBS) were purchased from Hylcone (Logan, UT, USA); echinacoside [high-performance liquid chromatography (HPLC)≥98%, 11167-200503, National Institutes for Food and Drug Control, China]; acteoside (HPLC≥98%, AB0497, ALFA, China); isoacteoside (HPLC≥98%, AB17021202, ALFA, China); 2’-acetyl acteoside (HPLC≥98%, PRF7081243, Chengdu Pu Rui Fa Technology, Co., Ltd. China); cistanoside A, cistanoside C, and 6’-acetyl acteoside (HPLC≥95%, isolated and purified in our lab) (Xu et al., 2017).

### Plant Materials and Preparation

Dried stems of *C. deserticola* Y. C. Ma were purchased from Yongning County, Ningxia province in September 2015, the specific coordinates of plant picking was (106.026597, 38.262816). The herb was identified by Prof. Ling Dong (department of pharmacognosy, Ningxia Medical University), with a voucher specimen (#20150901) was available in the herbarium of pharmaceutical analysis. A total of 10.0 kg dried and powdered *C. deserticola* was extracted by using heat reflux method, the detailed procedure was as follows: the solvent was 75% ethanol, the ratio of *C. deserticola* material to solvent was 1:8, the reflux time was lasted 2 h, and the materials were reflux for three times; then all of the filtrates were mixed together and concentrated under reduced pressure condition to afford 6.5 kg *C. deserticola* ethanol extracts (CDE). For animal experiment, 0.5% carboxymethyl cellulose sodium (CMC-Na) was used to suspend CDE, and the orally administered dosage was set as 200, 400, and 800 mg/kg/day, with a volume of 1 ml/100 g body weight of each animal; for western-blot analysis, dimethyl sulfoxide was employed to dissolve CDE and then diluted with DMEM to acquire the final concentration of 0.01, 0.1 1 mg/ml; for HPLC analysis, methanol was applied to dissolve CDE, followed by filtered and diluted with initial mobile phase to get the proper concentration.

### High-Performance Liquid Chromatography Analysis of *Cistanche deserticola* Extract

The major chemical constituents of CDE were identified by using HPLC with the corresponding reference substances. The chromatography conditions were set as follows: Agilent 1220 RP-HPLC instrument with C_18_ column (4.6 i.d. × 250 mm; 5 µm), a gradient elution mobile phase mainly contained water and acetonitrile was used to obtain well separated peaks: solvent A (water containing 0.5% acetic acid) and B (acetonitrile): 0–4 min, 85–83% A; 4–10 min, 83–80% A; 10–30 min, 80–75% A; 30–40 min, 75–70% A. The sample injection volume was 5 µl, the temperature was controlled at 30°C, and UV detection was performed at 333 nm (Dong et al., 2018). Reference substances including echinacoside, acteoside, isoacteoside, cistanoside A, cistanoside C, 2’-acetyl acteoside, and 6’-acetyl acteoside were used to identify the corresponding peak.

### Animal Experiments

For animal experiment, all procedures were performed in strict accordance with the protocol approved by the institutional animal care and use committee guide of Ningxia Medical University (NXMU-20130311). Forty-eight adult female Sprague-Dawley rats weighing about 237 ± 25 g were obtained (Ningxia Medical University, Yinchuan, China) and maintained for 1 week with a standard pellet diet and tap water under an environmentally controlled condition. Then, each rat underwent either anesthesia (chloral hydrate, 100 mg/kg, i.p.) only, sham ovariectomized (sham), or two ovaries were both removed and then randomly divided into five groups: model group (OVX) was orally administrated with 0.5% CMC-Na; positive group (EV) with 1 mg/kg/day of estradiol valerate; low (CDEL), moderate (CDEM), and high (CDEH) dosage groups with 200, 400, and 800 mg/kg/day of CDE, respectively. The experiments were lasted for 12 weeks; the body weight of each rat was measured biweekly with the administration dose adjusted accordingly. After the last day of the intervention, 24 h urine, serum, femora, tibia, and uterus were collected and stored at −80°C for further different analysis.

### Biochemical Parameters

The levels of serum SOD, GSH, MDA, cathepsin K, BGP, and urinary DPD were determined by the corresponding reagent kits, while the activity of tartrate-resistant acid phosphatase (TRAP) was evaluated according to a reference (Jiao et al., 2009). The activity of alkaline phosphatase (ALP) and the levels of Ca and P were estimated by employing an automatic analyzer machine (Ciba-Corning 550, USA).

### Bone Mineral Density and Micro-Computed Tomography Analysis

A dual-energy X-ray absorptiometry machine (Lunar, USA) was employed to estimate the bone mineral density (BMD, g/cm^2^) of the right femur of each rat with the scan mode was set as small animal. Then, the same femur was used to evaluated the 3D image of trabecular bone microarchitecture by applying a micro-CT scanner apparatus (GE, American), the region of interest (ROI) was chose by setting a same coordinates in the growth plate of the femur of each sample, the microarchitecture parameters including bone mineral content (BMC), tissue mineral content (TMC), tissue mineral density (TMD), trabecular separation (Tb. Sp), trabecular number (Tb. N), and trabecular thickness (Tb. Th) were obtained by analyzing the ROI.

### Western Blot Determination

Osteoclasts were induced by using RAW 264.7 cells added with MCSF (25 ng/ml) and RANKL (20 ng/ml) (Yang et al., 2019). After 6 days of lasted stimulated, the matured osteoclast cells which were identified for TRAP activity were treated with or without CDE (0.01, 0.1, and 1 mg/ml, respectively) for 48 h, then the cells were lysed by lysis buffer, and the supernatants were centrifuged and separated by 10% sodium dodecyl sulfate-polyacrylamide gel electrophoresis and transferred onto PVDF membranes. The membrane was blocked with 5% non-fat milk at ambient temperature for 1 h and then incubated with primaries antibodies against TRAF6 (1:400), RANKL (1:400), RANK (1:400), IKKβ (1:400), NFκBIA (1:400), OPG (1:400), PI3K (1:400), AKT (1:400), c-Fos (1:400), NFAT2 (1:400), β-actin, and GAPDH (1:1,000). The same membranes were stripped and probed again with corresponding antibodies, detected by the Image Lab Software at the end. Each of the experiment was repeated three times, with β-actin or GAPDH was an internal control.

### Statistical Analysis

The data of our experiments, described as the mean ± SD, were analyzed by using one-way analysis of variance followed by Dennett’s test (SPSS 22.0 software, SPSS, USA), *p* < 0.05 was considered as statistically significant.

## Results

### Main Chemical Constituents of *Cistanches deserticola* Extract

**Figure 1:**
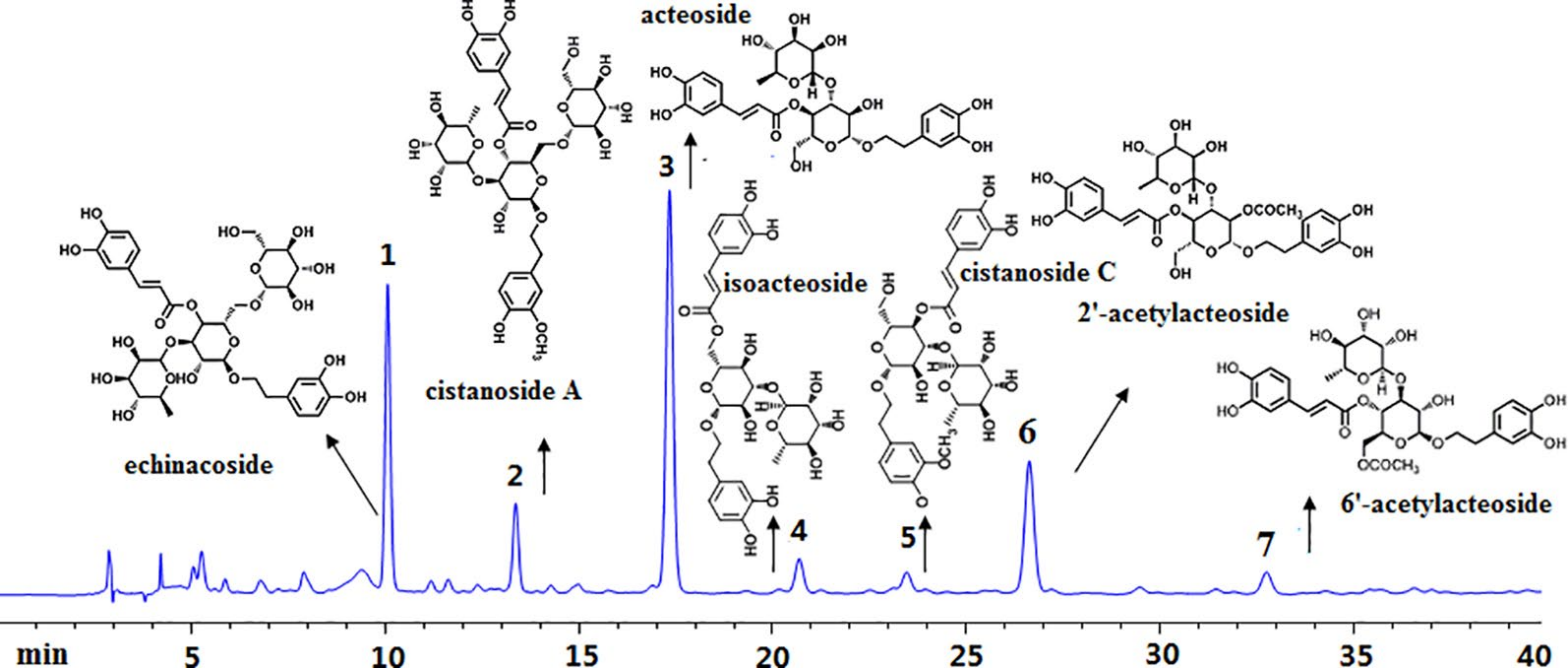
High-performance liquid chromatography analysis of *Cistanches deserticola* extract. Peaks were detected at 333 nm. The main constituents were calculated as 1-echinacoside, 2-cistanoside A, 3-acteoside, 4-isoacteoside, 5-cistanoside C, 6-2’-acetylacteoside, and 7-6’-acetylacteoside.

By using corresponding reference substances, seven mainly phenylethanoid glycosides compounds including echinacoside, cistanoside A, acteoside, isoacteoside, cistanoside C, 2’-acetyl acteoside, and 6’-acetyl acteoside were identified existing in this extract, their corresponding chromatography peaks and structures were showed in **Figure 1**.

### Effects of *Cistanches deserticola* Extract on the Body, Uterine, and Vagina Weights

**Figure 2:**
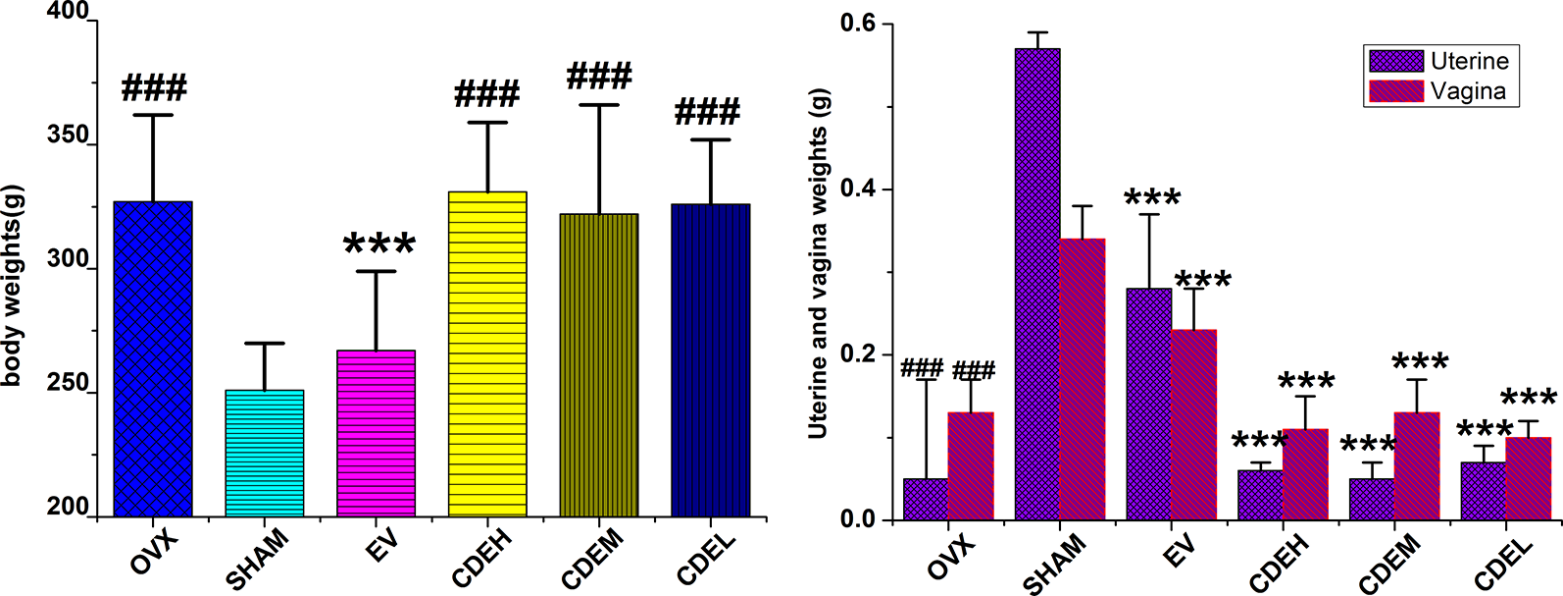
Effects of *Cistanches deserticola* extract or estradiol valerate on body, uterine, and vagina weights of rats (*n* = 8); data presented as mean ± SD, ^***^ *p* < 0.001 *versus* ovariectomized model group; **^###^** *p* < 0.001 *versus* sham group.

There was no significant difference in the initial mean body weight before surgery. However, 3 months after ovariectomized operation, the body weights of the rats in OVX model group were significantly increased by 35.7%, whereas the uterine and vagina wet weights were sharp declined by 91.2 and 61.8%, respectively, as compared to the sham rats (*p* < 0.001). CDE, with the dosage of 200–800 mg/kg/day, showed no influence on these noticeably increased body weights and decreased uterine and vagina wet weights, as the **Figure 2** showed. However, totally different to CDE, 1 mg/kg/day of EV exhibited a significant estrogenic effect, of which the gained body weight and decreased uterine and vagina weights in OVX rats were significantly reversed by EV supplemented.

### Effects of *Cistanches deserticola* Extract on Bone Mineral Density and Bone Microarchitecture

**Figure 3:**
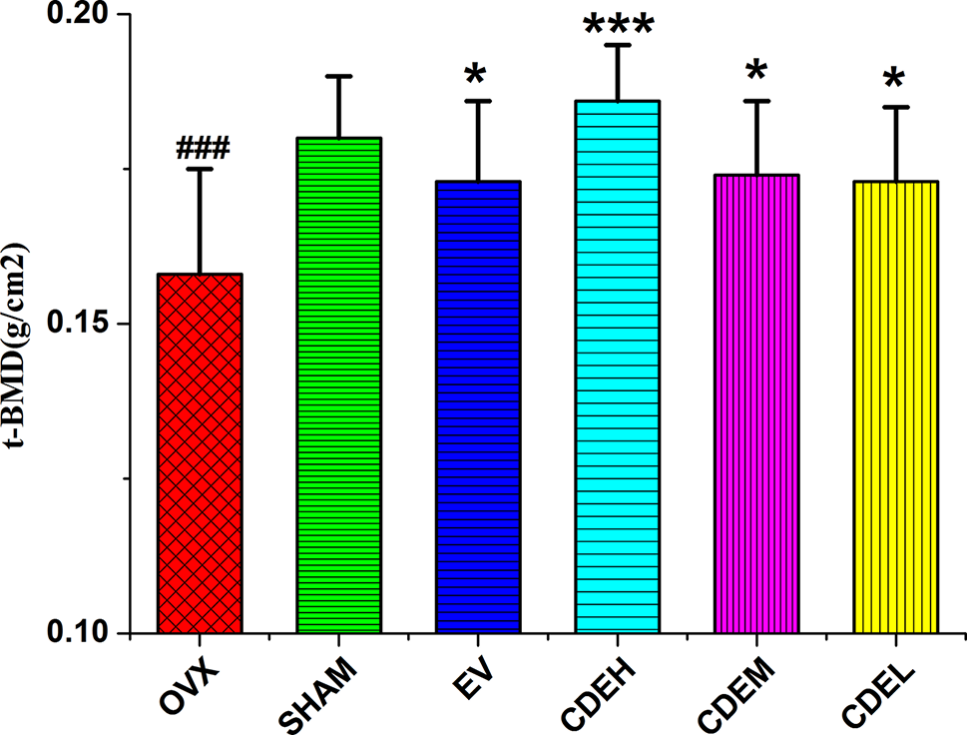
Effects of *Cistanches deserticola* extract or estradiol valerate on total bone mineral density of right femur of rats (*n* = 8); values were described as mean ± SD, ^*^ *p* < 0.05, ^***^ *p* < 0.001 *versus* ovariectomized model group; **^###^** *p* < 0.001 *versus* sham group.

An intuitively declining tendency was observed in OVX group with BMD was 0.158 ± 0.016 g/cm^2^ as compared to the sham rats with the BMD was 0.180 ± 0.010 g/cm^2^ (*p* < 0.001), which implied the BMD of the rats in OVX model group decreased about 12.2% after 12 weeks of the ovariectomized surgery as compared to the sham rats. As **Figure 3** showed, all of CDE treated rats showed an increased BMD by 15.1% (*p* < 0.001), 8.1% (*p* < 0.05), and 9.2% (*p* < 0.05), respectively, as compared to OVX model group.

**Figure 4:**
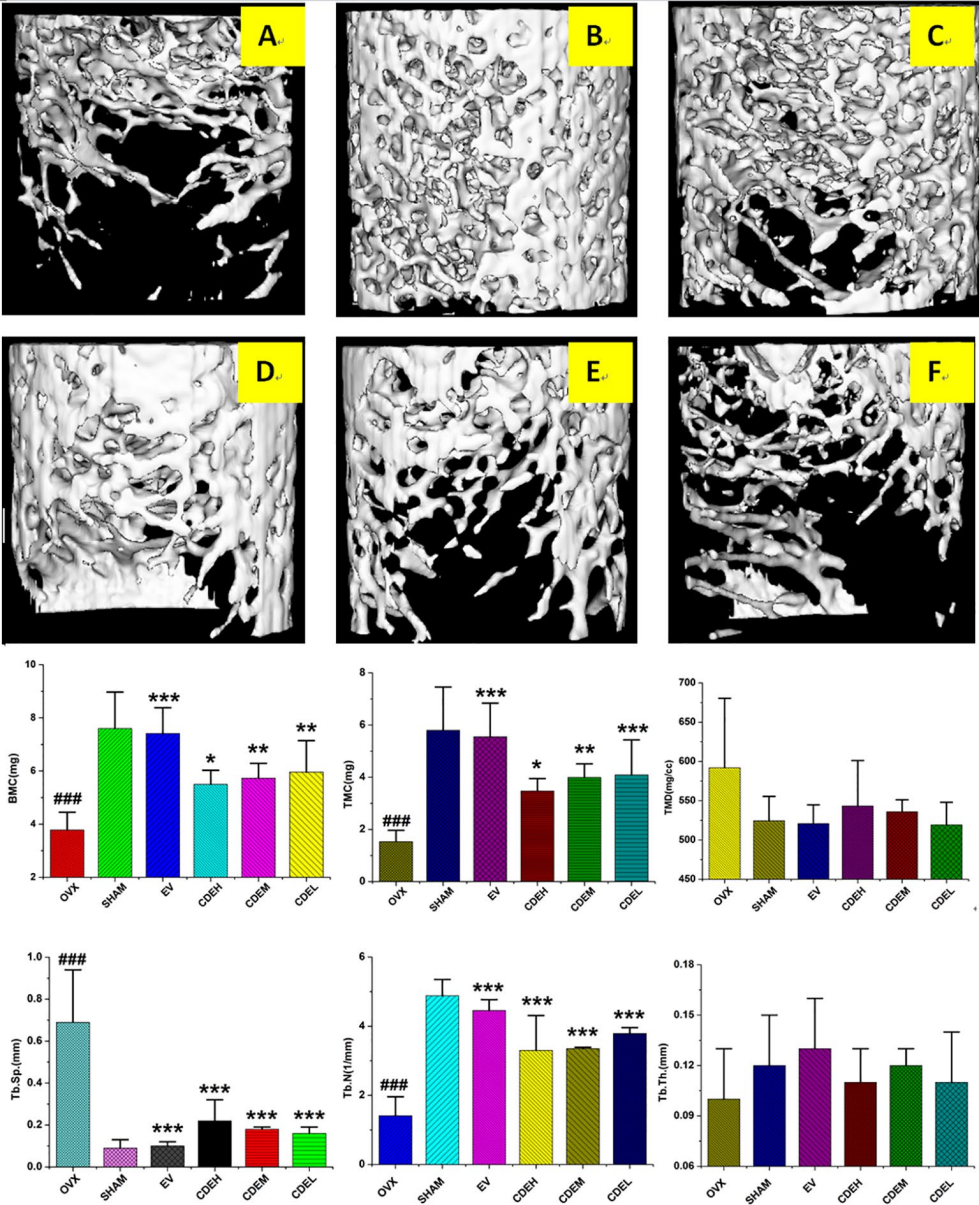
Micro-computed tomography scan images of microarchitecture of right femur of *Cistanches deserticola* extract (CDE) treated rats. The photographs shown were representative of four different rats in each group; **(A)** ovariectomized (OVX) group; **(B)** sham group; **(C)** estradiol valerate (EV) group; **(D)** high CDE group; **(E)** moderate CDE group; **(F)** low CDE group. The measured parameters were including bone mineral content (BMC), tissue mineral content (TMC), tissue mineral density (TMD), trabecular separation (Tb. Sp), trabecular number (Tb. N), and trabecular thickness mineral (Tb. Th). The OVX rats expressed a notable reduction of microarchitecture area and trabecular number. CDE and EV-treated reversed the above mentioned parameters at the same degree after 12 weeks treatment. Data were described as mean ± SD; *p < 0.05, **p < 0.01, ***p < 0.001 *versus* OVX model group; *^###^* *p* < 0.001 *versus* sham group.

The 3D-image and the corresponding quantitative results of trabecular bone microarchitecture of rats were showed in **Figure 4**. Consistent with the results of BMD, an obviously reduction of trabecular area was obtained in OVX model group as compared with the sham rats. However, the deterioration of trabecular bone was partly improved by CDE intervention, and except TMD and Tb. Th, the other trabecular bone parameters including BMC, TMC, and Tb. N were significantly increased and Tb. Sp was notably decreased after CDE treatment.

### Effects of *Cistanches deserticola* Extract on Urine and Serum Biochemical Parameters

**Figure 5:**
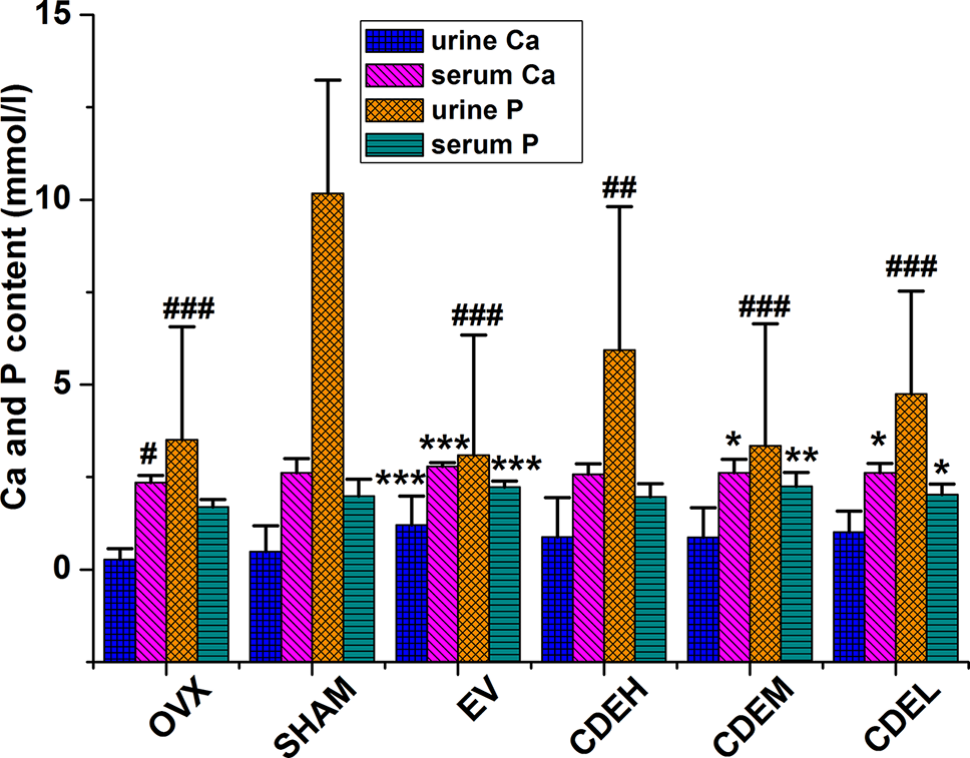
Effects of *Cistanches deserticola* extract or estradiol valerate on urine and serum Ca and P of rats (*n* = 8); data were expressed as mean ± SD, * *p* < 0.05, ** *p* < 0.01, *** *p* < 0.001 *versus* ovariectomized model group; **^##^** *p* < 0.01, **^###^** *p* < 0.001 *versus* sham group.

Twelve weeks after the ovariectomized operation, a declining but non-statistically trend of urinary excretion level of Ca was observed in OVX model group, as **Figure 5** showed, whereas the level of urine P in rats of the OVX model group was declined about three times less than the sham rats (*p* < 0.001). After treatment with CDE (200–400 mg/kg/day) for 12 weeks, the reduction of serum Ca and P were significantly prevented (*p* < 0.05), and the decreased urinary excretion level of P was also inhibited in all the CDE treated groups (*p* < 0.01).

### Effects of *Cistanches deserticola* Extract on Bone Formation and Resorption Markers

**Figure 6:**
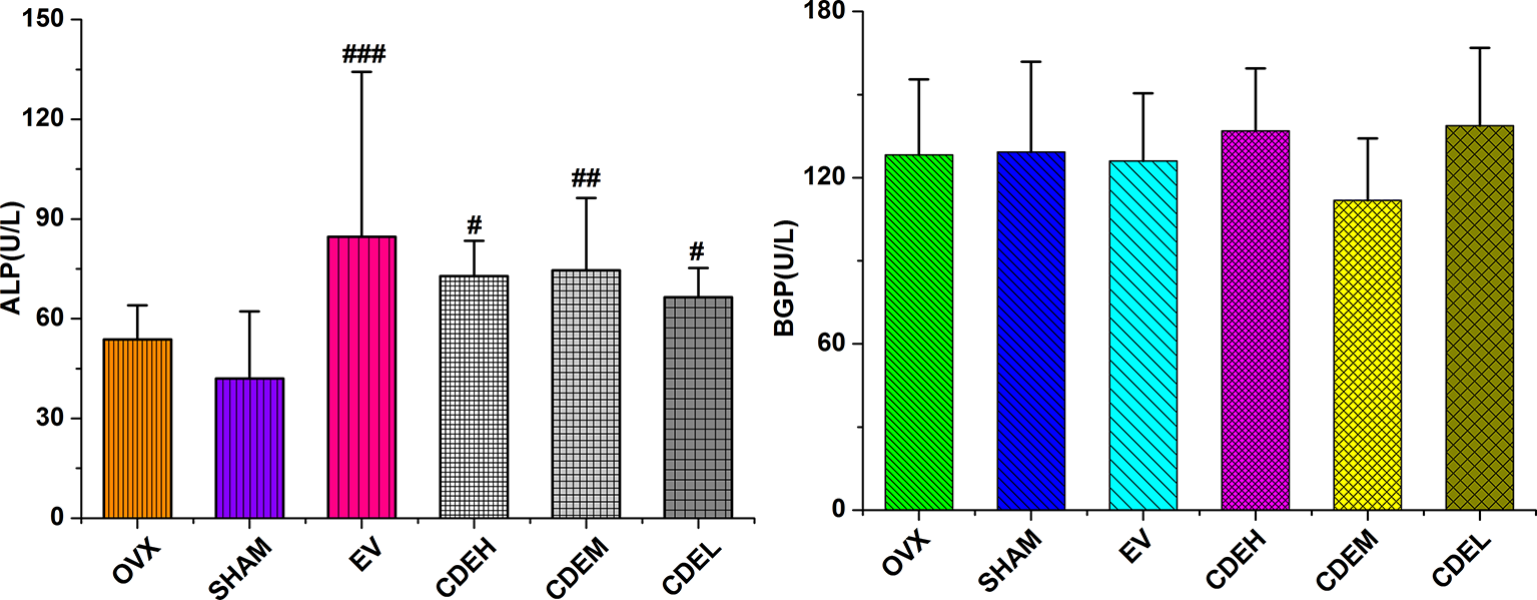
Effects of *Cistanches deserticola* extract or estradiol valerate on serum alkaline phosphatase and bone gla-protein activities of rats (*n* = 8); all values were described as mean ± SD. **^#^** *p* < 0.05, **^##^** *p* < 0.01, **^###^** *p* < 0.001 *versus* sham group.

**Figure 7:**
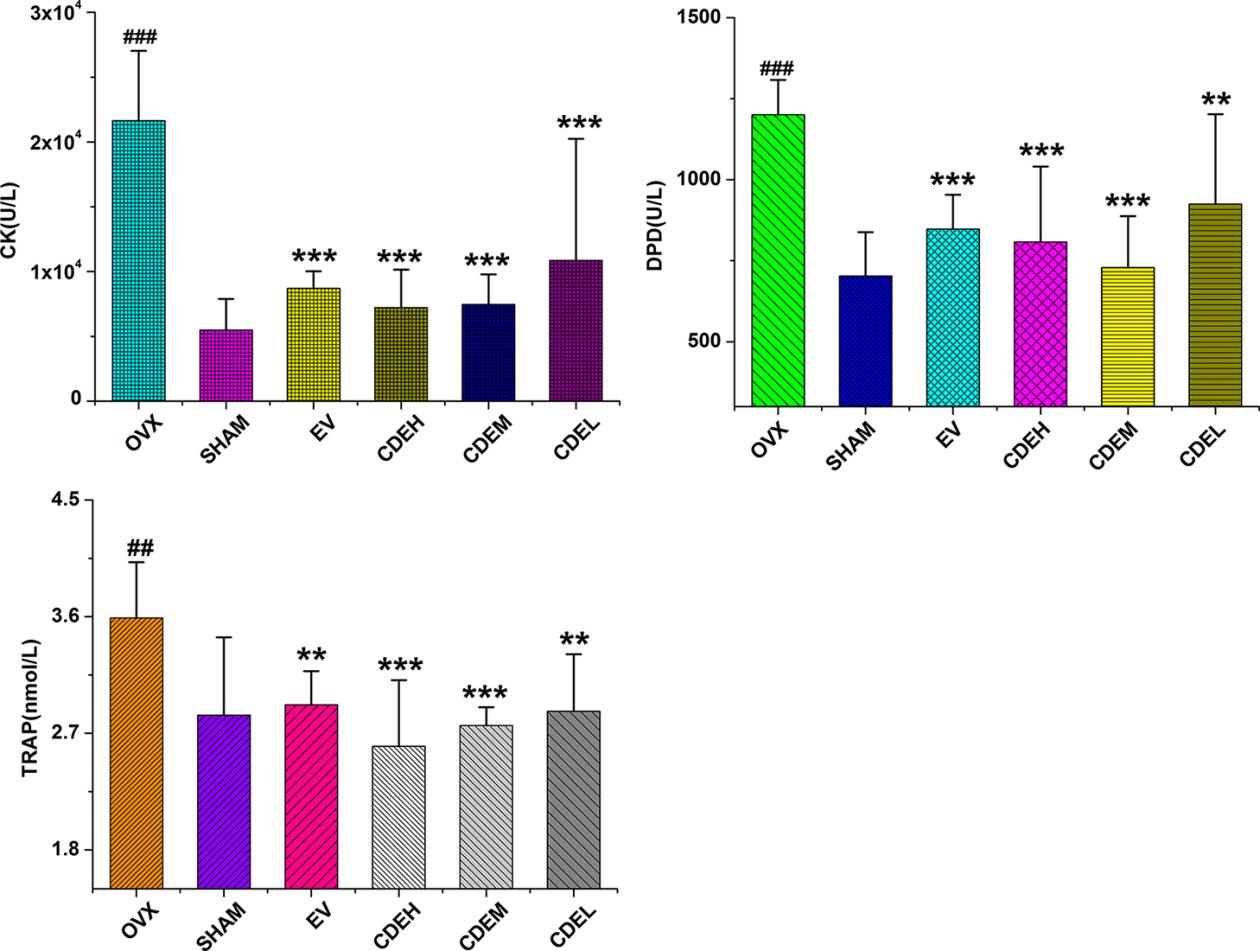
Effects of *Cistanches deserticola* extract or estradiol valerate on serum cathepsin K (CK), deoxypyridinoline, and tartrate-resistant acid phosphatase activities of rats (*n* = 8); all values were presented as mean ± SD, ^**^ *p* < 0.01, ^***^ *p* < 0.001 *versus* ovariectomized model group; **^##^** *p* < 0.01, **^###^** *p* < 0.001 *versus* sham group.

Concerning the bone formation markers of ALP and BGP (**Figure 6**), the activity of ALP, not BGP, was significant improved in all three doses of CDE (200–800 mg/kg/day) treated groups as compared to the sham rats. Concerning the bone resorption markers of TRAP, DPD, and cathepsin K (**Figure 7**), the activities of all the three bone resorption markers were significantly increased about 20.9∼74.8% in the rats of OVX model group as compared to the sham rats; and expectedly, CDE exhibited great potential in suppressing all of these indices, of which the activity of cathepsin K was decreased by 49.9∼66.7% (*p* < 0.001), the level of DPD was declined by 22.9∼39.3% (*p* < 0.01), and the property of TRAP was prevented by 20.1∼27.6% (*p* < 0.01), respectively, as compared with the rats of OVX model group.

### Effects of *Cistanches deserticola* Extract on Activities of Glutathione and Superoxide Dismutase as Well as Malondialdehyde Content

**Figure 8:**
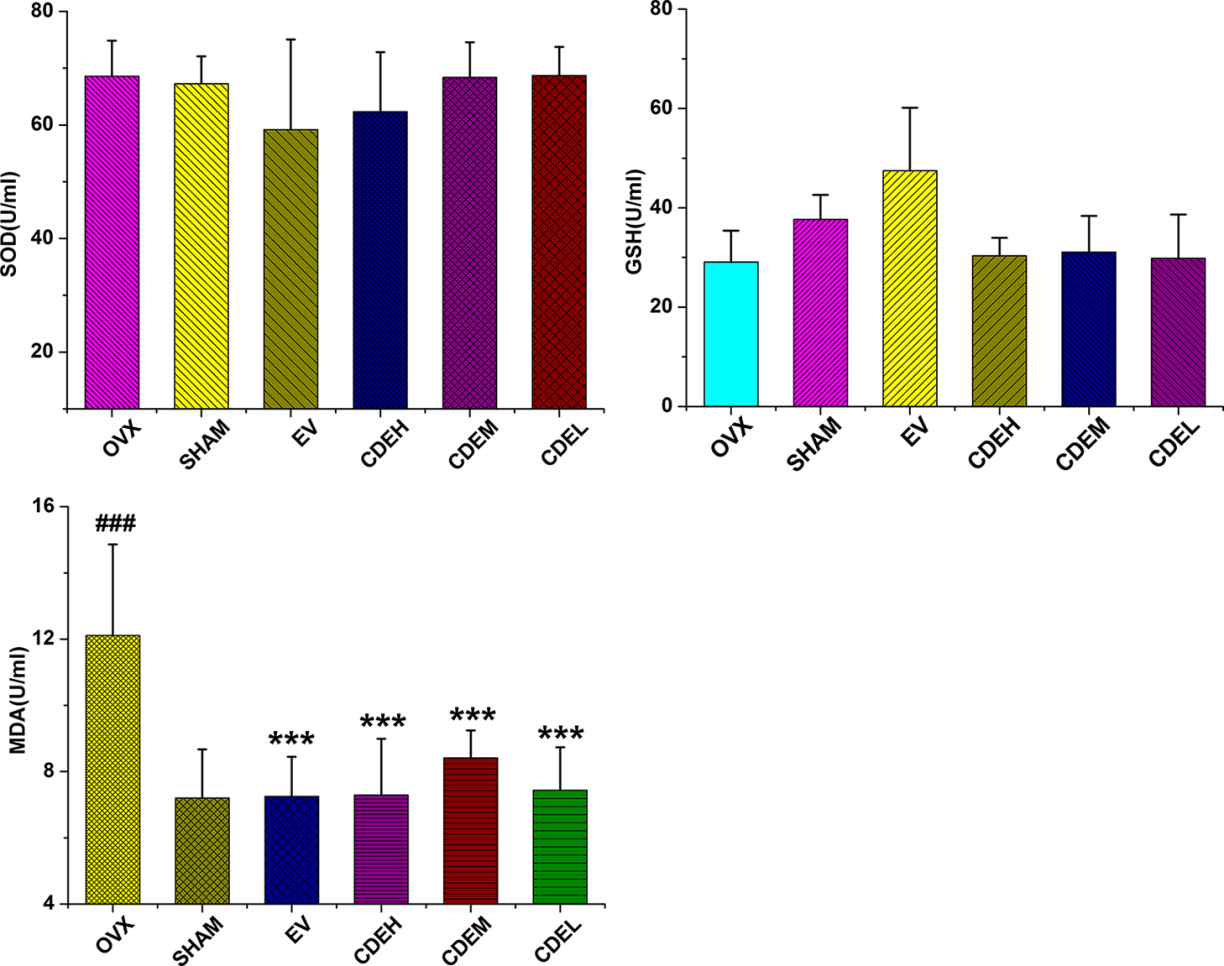
Effects of *Cistanches deserticola* extract or estradiol valerate on serum superoxide dismutase, glutathione, and malondialdehyde activities of rats (*n* = 8); all values were described as mean ± SD, ^***^ *p* < 0.001 *versus* ovariectomized model group; **^###^** *p* < 0.001 *versus* sham group.

There were no significant differences on the activities of SOD and GSH between the OVX and sham groups after 3 months of ovariectomized operation, as **Figure 8** showed, whereas the content of MDA was obviously enhanced in rats of OVX model group by 68.2% in comparison to the sham rats, and these increased MDA levels were significantly suppressed by low, moderate, and high dosage of CDE treated groups with the inhibition percentages were 66.1, 44.0, and 62.8%, respectively, as compared with the rats of OVX model group.

### Effects of *Cistanches deserticola* Extract on Protein Expression Levels of TRAF6, RANKL, RANK, NFκBIA, IKKβ, PI3K, AKT, OPG, c-Fos, and NFAT2

**Figure 9:**
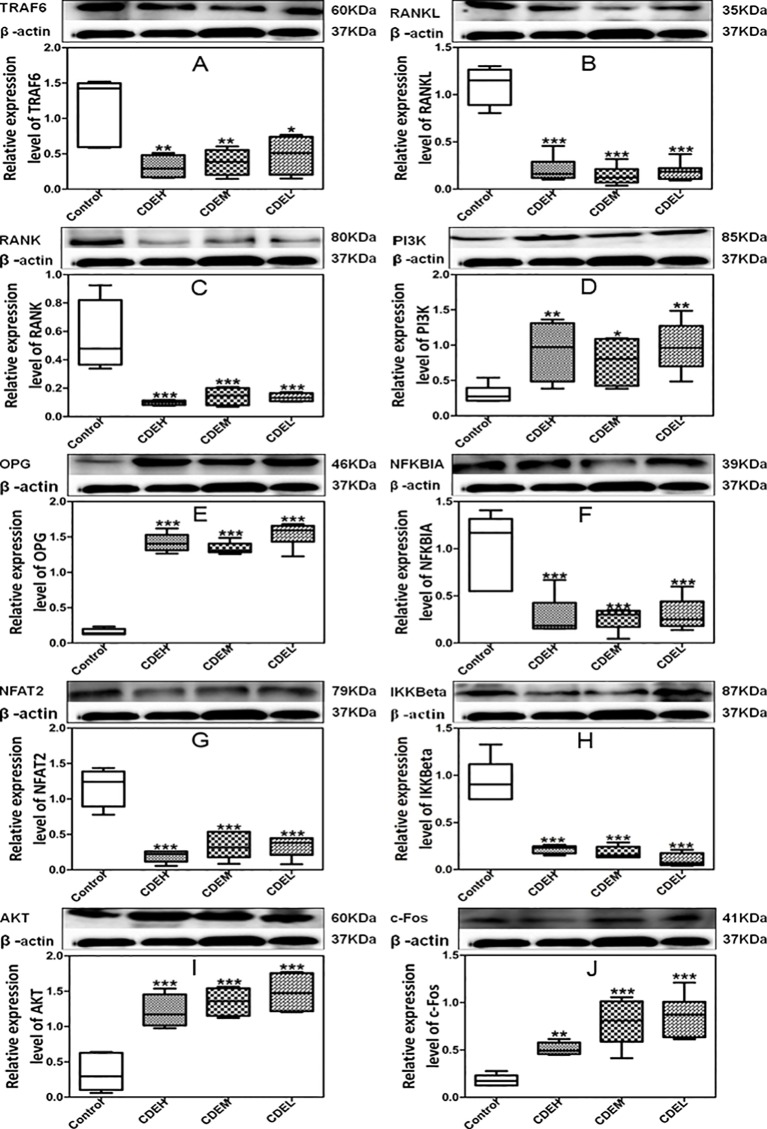
Effects of *Cistanches deserticola* extract (CDE) on the protein expressions of TRAF6 **(A)**, RANKL **(B)**, RANK **(C)**, PI3K **(D)**, OPG **(E)**, NFKBIA **(F)**, NFAT2 **(G)**, IKKβ **(H)**, AKT **(I)**, and c-Fos **(J)** (n = 3); high CDE (cell treated with 1 mg/ml of CDE), moderate CDE (cell treated with 0.1 mg/ml of CDE), low CDE (cell treated with 0.01 mg/ml of CDE), control (cell treated without CDE); the proteins expressions were normalized to β-actin, and quantitative data of every signal protein was showed as percentage of the value of control. All values were described as mean ± SD. *p < 0.05, **p < 0.01, ***p < 0.001 *versus* control group.

**Figure 10:**
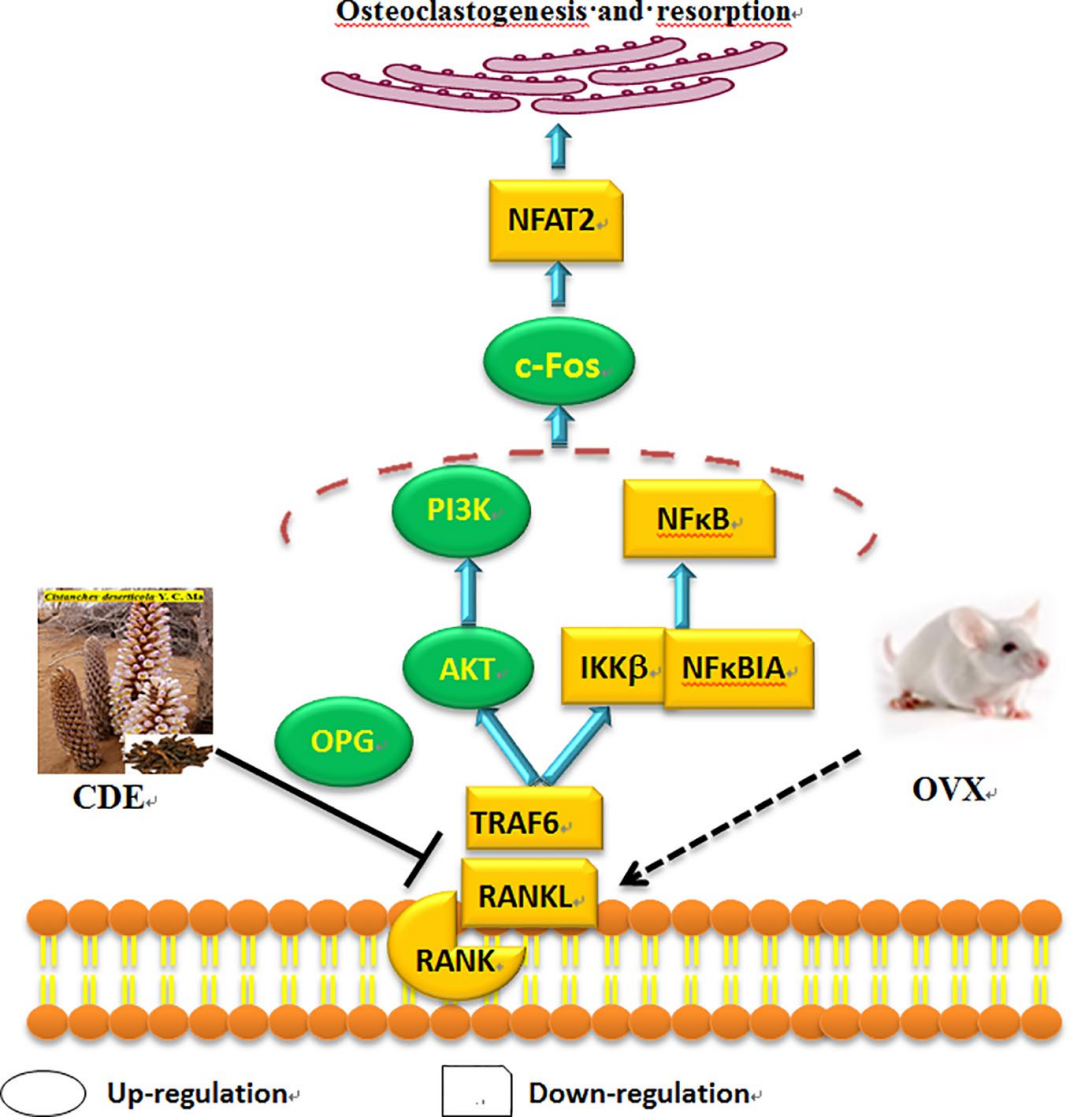
Hypothesized molecular mechanism: *Cistanches deserticola* extract (CDE) could prevent bone loss on ovariectomized rats through RANKL/RANK/TRAF6-induced inactivation of NF-κB and activation of PI3K/AKT pathways as well as c-Fos stimulation and NFAT2 suppression, which evidenced by the expression levels of TRAF6, RANKL, RANK, NFκBIA, NFAT2, and IKKβ were down-regulated, whereas OPG, c-Fos, AKT, and PI3K were significantly up-regulated by CDE treatment as compared to control group.

As **Figure 9** showed, the expressions of TRAF6, RANKL, RANK, IKKβ, NFκBIA, and NFAT2 were significantly down-regulated whereas the levels of PI3K, AKT, OPG, and c-Fos were obviously up-regulated after treatment with CDE as compared to the control. Hypothesized mechanism (**Figure 10**) by which CDE could down-regulate the levels of RANKL, RANK, and TRAF6 and up-regulate the expression of OPG, thus the binding quantities of RANKL with RANK were reduced; consequently, the downstream pathways including NF-κB signal was suppressed and PI3K/AKT was stimulated, and these signaling cascade together led the activation of c-Fos was promoted and NFAT2 was inhibited, and finally the osteoclastic bone resorption was prevented.

## Discussion

Nowadays, natural extracts and compounds with anti-osteoporotic activity but few side effects have attracted the interests of plenty pharmaceutical scientists. TCM, for more than a millennium, have been extensively used, apparently safely and effectively, to alleviate various symptoms of diseases including osteoporosis (Lin et al., 2017). It was found TCM that traditionally used to invigorate and keep kidney essence, were also can be used to treat osteoporosis, including *Epimedium brevicornu* Maxim., *Eucommia ulmoides* Oliver., *Cuscuta chinensis* Lam., *Dipsacus asper* Wall. ex Henry, etc. (Yang et al., 2011; Tao et al., 2017; Qi et al., 2018). Numbers of antiosteoporotic bioactive compounds, including polyphenol and phenylethanoid glycosides, were identified and isolated from dozens of natural medicinal herb (Ma et al., 2015; Ye et al., 2015; Xu et al., 2017; Liu et al., 2018).

*C. deserticola* is an important natural health food and classic TCM has long been used as a common tonic in China and Japan. It is found possessed a favorable safety profile and broad medicinal functions for the treatment of kidney deficiency, muscle debility, and lumbar weakness. According to the theory of TCM and our previous experiments (Liu et al., 2018), TCM that traditionally used to invigorate kidney was generally applied to prevent osteoporosis, therefore indicating *C. deserticola* maybe possess anti-osteoporotic property. Several published experimental studies had discovered the therapeutic effect of *C. deserticola* extracts and *C. deserticola* polysaccharide as well as *Cistanche* herb on osteoporosis (Liang et al., 2011; Li et al., 2012; Liang et al., 2013; Song et al., 2018), However, the detailed mechanisms of anti-osteoporotic effect of CDE, especially the upstream signaling, were remain unclear. Now, in the present study, an OVX rat model which could simulate postmenopausal osteoporosis was used to investigate the anti-osteoporosis action of *C. deserticola in vivo*, and the proteins levels of RANKL, RANK, and TRAF6 as well as other key regulators related to bone resorption were also analyzed to estimate the possible mechanisms.

OVX induced postmenopausal osteoporosis accompanied by a sharp decline in bone quality, bone microarchitecture, uterine, and vagina wet weights, as well as an obvious enhancement in bone resorption and body weight, of which were in part due to estrogen lost (Nian et al., 2009). In the present *in vivo* experiment, CDE showed potent anti-osteoporosis activity evidenced by the enhanced total BMD and improved trabecular bone microarchitecture, and the increased body weight and decreased uterine and vagina weights of the OVX rats were not affected by CDE treatment, but significantly reversed by EV administration, which implied that CDE could enhance the bone formation without inducing the side effects on body and uterine organic tissues.

BMD is generally used in clinical for the diagnosis of osteoporosis and low level of BMD is considered as a major risk for bone fractures (Kanis et al., 2015). Besides the BMD, numerous lab experiments and clinical trials revealed that bone microarchitecture, especially the trabecular microarchitecture profile was also significantly contributed to bone quality (Le Corroller et al., 2013), thus makes the micro-CT 3D-image evaluation are meaningful in osteoporosis diagnosis nowadays. Our previous study revealed that OVX resulted in a sharp decline in content of BMC, TMC, Tb. N, and Tb. Th, and increase in level of Tb. Sp (Ma et al., 2015). In the present research, the bone trabecular microarchitecture structural parameters obtained by analyzing the micro-CT 3D-image of ROI showed CDE was effective in ameliorating trabecular microarchitecture and thus enhanced bone quality.

Furthermore, the contents of Ca and P of OVX rats were also employed to reflect the anti-osteoporotic effect. However, totally different from the previous published studies which the urinary excretion levels of Ca and P in OVX rats were signiﬁcantly increased after the ovariectomized surgery as compared to sham rats (Nian et al., 2009), our data demonstrated that the level of P in urine of OVX rats was obviously declined (*p* < 0.001), almost three times less than the rats of sham. To analyze this diametrically opposed phenomenon, the levels of Ca and P in serum of OVX rats can not be ignored. It was discovered that although the urinary excretion level of P was significantly decreased in OVX rats, the serum Ca and P levels also showed non-statistically significant declining trends, those both decreased trends maybe ultimately resulted in a bone loss. Expectedly, CDE significantly increased serum Ca and P levels as well as decreased urinary P excretion induced by ovariectomized surgery, which further proved the fact that CDE could prevent bone loss.

Concerning the effect of CDE on bone metabolic index including ALP, BGP, TRAP, DPD, and cathepsin K, similar to the published data that ALP activity in OVX model group showed a non-statistically increasing trend which indicating an accelerated rate of bone turnover (Swaminathan, 2001) in postmenopausal osteoporosis, CDE treatment showed significant stimulation on ALP activity and also suppression on TRAP, DPD, and cathepsin K properties, implied that the therapeutic effect of CDE on OVX rats were mainly through inhibiting bone resorption.

Ovariectomized surgery was proved generally caused oxidative stress and impaired bone antioxidant system in adult rats (Muthusami et al., 2005). In our manuscript, OVX induced a high MDA level in rats. However, the content of MDA in OVX rats were significantly decreased by CDE intervention as compared to the model group (*p* < 0.001) which indicated the antioxidant activity of this herb. According to the Chinese “yin-yang” and “kidney dominate bone” theory, tonic herbs generally possessed antioxidant activity, and this property always correlated well with the amount of phenolic compounds exist in plants (Ou et al., 2003). And in our present study, a HPLC method was used to discover the phenolic compounds and other chemicals responsible for the anti-osteoporotic activity of CDE. Seven main phenylethanoid glycosides components including echinacoside, cistanoside A, acteoside, isoacteoside, acteoside C, 2’-acetylacteoside, and 6’-acetylacteoside were found in this extract. The structures of the above seven phenylethanoid glycosides were all rich in phenolic hydroxyl groups. Therefore, both the published and our experimental data revealed a plenty of phenolic compounds in this plant, all of which supported the antioxidant property of *C. deserticola*, thus may be could prevent bone loss by improving the bone antioxidant system. However, the structure-activity relationship between the different phenylethanoid glycosides and their antioxidant properties was still unclear. Only the number of phenolic hydroxyl groups can not totally reflect the anti-osteoporotic activities of phenylethanoid glycosides in *C. deserticola*, the other factors including the compact, ramification, and symmetric of structures also influenced the effective of the compounds (Chen et al., 2016; Chen et al., 2018a; Chen et al., 2018b; Teng et al., 2018; Chen et al., 2019), thus need us to estimate the related structure-activity relationship in our further studies.

As osteoporosis was mainly caused by an excessive osteoclastic bone resorption, the factors and regulators which related with the activation and differentiation of osteoclast were believed as crucial targets for preventing bone loss. RANKL, a member of tumor necrosis factor (TNF) family which was expressed in osteoclast precursors, could regulate the differentiation, survival, and activation of osteoclasts when it binds to its receptor RANK, and this process can not be initiated until TRAF6 was recruitment. In our manuscript, the expressions of RANKL, RANK, and TRAF6 were significantly down-regulated and the OPG was up-regulated by CDE treatment, it could be expected that the binding quantities of RANKL with RANK was decreased, together with the limited amounts of TRAF6 could be recruited, thus the differentiation and activation of osteoclasts were suppressed and the bone resorption was inhibited. The binding of RANKL/RANK/TRAF6 triggered a series of downstream signaling cascades including NF-κB and PI3K/AKT pathways. NF-κB was proved essential for osteoclastogenesis as the disruption of NF-κB could lead to an impaired osteoclast differentiation with an osteopetrotic phenotype (He et al., 2012), and NF-κB up-regulated c-Fos and down-regulated NFAT2 expressions during RANKL/RANK/TRAF-induced osteoclastogenesis (Takatsuna et al., 2005). c-Fos regulated expressions of NFAT2 in osteoclasts to mediate its osteoclastogenic effects, and NFAT2 conversely mediated RANKL-induced osteoclast formation, and its over-expression could rescue osteoclastogenesis in c-Fos cells (Boyce et al., 2005). Our data showed that the expressions of IKKβ, NFκBIA, and NFAT2 were down-regulated whereas the level of c-Fos was up-regulated by CDE treatment, indicating that CDE significantly suppressed RANKL/RANK/TRAF-induced NF-κB stimulation, and the activation of c-Fos was promoted and NFAT2 was consequently inhibited. Summarily, the therapeutic effect of CDE on OVX rats main through preventing RANKL/RANK/TRAF-induced NF-κB activation and PI3K/AKT inactivation as well as c-Fos stimulation and NFAT2 suppression, and finally the differentiation of osteoclast was inhibited and the corresponding bone resorption was suppressed.

## Ethics Statement

For the animal experiment, all procedures were performed in strict accordance with the protocol approved by the institutional animal care and use committee guide of Ningxia Medical University.

## Author Contributions

X-QM and YJ conceived the experiments. BZ, S-QD and L-LY performed the animal experiments. J-JL performed the western-blot tests. Y-HD wrote the draft of manuscript. Y-TL, NL, and X-JZ analyzed the data. C-LH revised the manuscript. All authors reviewed the manuscript.

## Funding

This work was supported by the grants from the National Natural Science Foundation of China (No.81560684); Science Technology Foundation of Higher Education of Ningxia (NGY2017090); Ningxia key research and invention program of science and technology cooperation of the East and the West (No. 2017BY084, 2017BY079); the West Light Foundation of the Chinese Academy of Sciences-Young Scientists of West 2017.

## Conflict of Interest

The authors declare that the research was conducted in the absence of any commercial or financial relationships that could be construed as a potential conflict of interest.

## Abbreviations

AKT, protein kinase B; ALP, alkaline phosphatase; BGP, bone gla-protein; BMC, bone mineral content; BMD, bone mineral density; CDE, *Cistanche deserticola* ethanol extracts; CK, cathepsin K; DMEM, Dulbecco’s modified eagle’s medium; DPD, deoxypyridinoline; EV, estradiol valerate; FBS, fetal bovine serum; GSH, glutathione; IKKβ, IκB kinase β; MCSF, macrophage colony-stimulating factor; MDA, malondialdehyde; NFAT2, nuclear factor of activated T cells c2; NF-κB, nuclear factor kappa B; OPG, osteoprotegerin; OVX, ovariectomized; PBS, phosphate buffered saline; PI3K, phosphatidylinositol 3-kinase; PVDF, polyvinylidene fluoride; RANK, receptor activator of nuclear factor kappa B; RANKL, receptor activator of nuclear factor kappa B ligand; ROI, region of interest; SOD, superoxide dismutase; Tb. N, trabecular number; Tb. Sp, trabecular separation; Tb. Th, trabecular thickness; TCM, traditional Chinese medicine; TMC, tissue mineral content; TMD, tissue mineral density; TNF, tumor necrosis factor; TRAF6, tumor-necrosis factor receptor-associated factor 6; TRAP, tartrate-resistant acid phosphatase.
